# Final report of a phase II study of interleukin 2 and interferon alpha in patients with metastatic melanoma.

**DOI:** 10.1038/bjc.1995.256

**Published:** 1995-06

**Authors:** W. H. Kruit, S. H. Goey, F. Calabresi, A. Lindemann, R. A. Stahel, H. Poliwoda, B. Osterwalder, G. Stoter

**Affiliations:** Department of Medical Oncology, Rotterdam Cancer Institute, The Netherlands.

## Abstract

Fifty-seven patients with metastatic melanoma were treated with interleukin 2 (IL-2) 7.8 MIU m-2 day-1 as a continuous infusion for 4 days combined with interferon alpha (IFN-alpha) 6 MIU m-2 day-1 subcutaneously on days 1 and 4. The cycle was repeated every 2 weeks for a maximum number of 13 cycles. Of the 51 evaluable patients, one (2%) achieved a complete and seven (14%) a partial response (total response rate 16%; CI 7-29%). Median time to progression and median survival were 2.5 and 11.3 months respectively. This regimen of IL-2 and IFN-alpha appeared to be only moderately active.


British Journal of Cancer (1995) 71, 1319-1321

© 1995 Stockton Press All rights reserved 0007-0920/95 $12.00

### Final report of a phase II study of interleukin 2 and interferon α in patients with metastatic melanoma

WHJ Kruit[1], SH Goey[1], F Calabresi[2], A Lindemann[3], RA Stahel[4], H Poliwoda[5], B Osterwalder[6] and G Stoter[1]

[1]*Department of Medical Oncology, Rotterdam Cancer Institute, PO Box 5201, 3008 AE Rotterdam, The Netherlands;* [2]*Department of Medical Oncology, National Cancer Institute Regina Elena, Viale Regina Elena 291, 00161 Rome, Italy;* [3]*Department of Hematology and Oncology, University Hospital, Hugstetter Str 55, D-79106 Freiburg, Germany;* [4]*Division of Oncology, Department of Medicine, University Hospital, CH-8091 Zürich, Switzerland;* [5]*Department of Hematology and Oncology, University Medical Center, D-3000 Hannover 61, Germany;* [6]*International Clinical Research Oncology, F Hoffmann-La Roche Ltd, Grenzacherstrasse 124, 4002 Basle, Switzerland.*

**Summary** Fifty-seven patients with metastatic melanoma were treated with interleukin 2 (IL-2) 7.8 MIU $m^{-2}$ $day^{-1}$ as a continuous infusion for 4 days combined with interferon α (IFN-α) 6 MIU $m^{-2}$ $day^{-1}$ subcutaneously on days 1 and 4. The cycle was repeated every 2 weeks for a maximum number of 13 cycles. Of the 51 evaluable patients, one (2%) achieved a complete and seven (14%) a partial response (total response rate 16%; CI 7-29%). Median time to progression and median survival were 2.5 and 11.3 months respectively. This regimen of IL-2 and IFN-α appeared to be only moderately active.

**Keywords:** interleukin 2; interferon alpha; immunotherapy; metastatic melanoma

Immunotherapy with recombinant interleukin 2 (IL-2) has been reported to yield a 5-27% response rate in metastatic melanoma (Rosenberg *et al.*, 1989*a*, 1993; Parkinson *et al.*, 1990; Whitehead *et al.*, 1991; Sparano *et al.*, 1993). Interferon α (IFN-α) alone in this group of patients has shown response rates of 12-22% (Robinson *et al.*, 1986; Kirkwood, 1991).

Based on the synergistic activity of IL-2 and IFN-α in preclinical experiments (Brunda *et al.*, 1987; Cameron *et al.*, 1988; Iigo *et al.*, 1988) and on the encouraging results of early clinical trials with this combination (Budd *et al.*, 1989; Lee *et al.*, 1989; Rosenberg *et al.*, 1989*b*), we decided to perform a phase II study. Here, we report the final analysis after a median follow-up period of 10.5 months (range 1.1-47+ months).

### Materials and methods

### *Patients*

Fifty-seven patients with metastatic melanoma were entered in the study. Eligibility criteria included: age 18-70 years, Karnofsky performance status 60-100, no metastases in the central nervous system, no significant cardiovascular history, normal pulmonary function, serum bilirubin and creatinine within normal range, normal bone marrow function (haematocrit > 30%, white blood count > 4000 $ml^{-1}$, platelets > 100 000 $ml^{-1}$), normal coagulation parameters, normal serum calcium and negative tests for HIV antibody and hepatitis B antigen.

Previous treatment with IL-2 or IFN-α was not allowed. Prior radiotherapy or chemotherapy had to be completed at least 4 weeks before entry into the study. Corticosteroids were prohibited.

The protocol was reviewed and approved by the institutional review board and the ethical committee of each participating centre.

Six patients were ineligible: three had non-measurable disease, two had brain metastases, one was pretreated with interferon 2β. Fifty-one patients were evaluable for response and toxicity. The patient characteristics are shown in Table I. The median time from initial diagnosis to immunotherapy was 24 months (range 1-142 months).

### *Treatment*

Patients were treated with IL-2 at a dose of 7.8 MIU $m^{-2}$ $day^{-1}$ by continuous infusion on days 1-4 and with IFN-α-2a 6 MIU $m^{-2}$ $day^{-1}$ by subcutaneous injection on days 1 and 4 of each treatment cycle. IL-2 (Teceleukin) and IFN-α (Roferon-A) were supplied by Hoffmann-LaRoche, Basle, Switzerland. Cycles were repeated every 2 weeks.

Evaluation of response was performed after 4 cycles and every 2 months thereafter. Patients who responded or experienced no change received nine additional treatment cycles. Further continuation of treatment beyond 6 months was allowed.

**Table I** Patient characteristics

| | |
|---|---|
| Number of patients | 51 |
| Age (years) | |
| Median | 49 |
| Range | 21-72 |
| Sex | |
| Male | 29 (57%) |
| Female | 22 (43%) |
| Performance status (Karnofsky) | |
| Median | 90 |
| Range | 70-100 |
| Prior therapy | |
| None | 25 (49%) |
| Chemotherapy | 19 (37%) |
| Radiotherapy | 5 (10%) |
| Hormone therapy | 2 (4%) |
| Distribution of metastatic sites | |
| Lung | 20 (39%) |
| Lymph nodes | 29 (57%) |
| Skin | 16 (31%) |
| Liver | 17 (33%) |
| Bone | 10 (20%) |
| Number of metastatic sites | |
| 1 | 15 (29%) |
| 2 | 14 (27%) |
| 3 | 10 (20%) |
| 4 | 9 (18%) |
| 5 | 2 (4%) |
| 6 | 1 (2%) |

Correspondence: WHJ Kruit
Received 9 November 1994; revised 10 January 1995; accepted 12 January 1995

### Monitoring

Toxicity was recorded and analysed using the WHO grading system (WHO, 1979). Side-effects not described in the WHO guidelines were graded from mild (grade 1) to life-threatening (grade 4).

Response was evaluated according to the WHO guidelines (WHO, 1979). A complete response (CR) was defined as the disappearance of all known disease for at least 4 weeks. A partial response (PR) was defined as a reduction in the sum of the products of the largest perpendicular diameters of the tumour lesions by at least 50% for more than 4 weeks. Stable disease (SD) denoted less than 50% tumour reduction and less than 25% tumour progression. Progressive disease (PD) was defined as the appearance of a new lesion or an increase in size of more than 25% in any lesion.

### Results

### Response

Of the 51 eligible patients, 24 (47%) received 2–4 treatment cycles, 12 (24%) 5–8 cycles, 13 (26%) 9–13 cycles, one patient 15 and one patient 16 cycles. Four patients were taken off study early, one because of intercurrent illness and three because of grade 4 toxicity.

The overall response rate was 16% (95% confidence interval 7–29%), including one CR (2%) and seven PRs (14%). Twenty patients (39%) had stable disease. In 23 (45%) patients progressive disease was documented. Three of the responders were male and five were female. Responses were seen in skin lesions (36%), lymph nodes (27%), lung (18%) and liver (18%). Of note, bone metastases did not respond. All responses occurred in the first 3 months of treatment.

The median duration of response was 8.2 months (range 4.5–39+ months). For all 51 patients the median time to progression was 2.5 months (range 0.5–39+ months). Time to progression for responding patients was 8.2 months (range 4.5–39+ months), for patients with stable disease 3.6 months (range 1.7–9.4 months) and for progressive disease patients 1.2 months (range 0.5–2.0 months). The median survival of all patients was 11.3 months (Figure 1), and of the responding patients 20.2 months.

### Toxicity

An overview of the observed toxicity is presented in Table II. Frequently occurring side-effects were fever, skin rash, nausea, vomiting, diarrhoea and malaise. Two-thirds of patients had tachycardia and hypotension, mostly of mild to moderate grade. Life-threatening hypotension requiring vasopressors occurred in three patients, who were taken off study (see above). One patient developed ventricular extrasystoles and another patient atrial fibrillation. In a minority of patients neurological abnormalities and mental disturbances were seen. Neurotoxicity included aphasia, peripheral neuropathy, somnolence, confusion and agitation.

Two patients required dose reductions because of adverse events, and in eight patients short interruption of treatment was needed. Not toxic death occurred and all toxicities resolved after cessation of immunotherapy. Chronic cumulative fatigue occurred after about 3 months of treatment. Consequently, only two patients received more than 13 cycles.

The most frequent manifestation of haematological toxicity was anaemia (71%). Thrombocytopenia was seen in 18% of the patients. Moderate and reversible increases in serum creatinine and bilirubin occurred in a minority of patients.

**Figure 1** Survival curve (median survival 11.3 months).

**Table II** Adverse events

| Adverse events | Number of patients (%) | 1 | 2 | 3 | 4 |
|---|---|---|---|---|---|
| Fever | 51 (100) | 0 | 21 | 30 | 0 |
| Skin rash erythema | 36 (71) | 13 | 19 | 4 | 0 |
| Nausea vomiting | 48 (94) | 7 | 28 | 13 | 0 |
| Diarrhoea | 38 (75) | 10 | 20 | 8 | 0 |
| Malaise | 29 (57) | 4 | 15 | 10 | 0 |
| Weight gain | 15 (30) | 13 | 2 | 0 | 0 |
| Hypotension | 39 (76) | 7 | 18 | 11 | 3 |
| Tachycardia | 36 (71) | 13 | 20 | 3 | 0 |
| Dyspnoea | 10 (20) | 4 | 4 | 2 | 0 |
| Mental disturbances | 8 (16) | 5 | 3 | 0 | 0 |
| Creatinine | 19 (37) | 16 | 3 | 0 | 0 |
| Alkaline phosphatase | 30 (59) | 12 | 15 | 3 | 0 |
| Bilirubin | 9 (18) | 7 | 2 | 0 | 0 |
| Anaemia | 36 (71) | 17 | 14 | 5 | 0 |
| Thrombocytopenia | 9 (18) | 7 | 2 | 0 | 0 |

### Discussion

In this study the combined use of IL-2 and IFN-α in the treatment of metastatic melanoma resulted in a 16% response rate, including 2% complete responses. These results are disappointing and not better than can be expected of conventional chemotherapy or immunotherapy with IL-2 alone.

Response rates of 21–44% have been reported in some studies using the combination of both cytokines (Lee *et al.*, 1989; Rosenberg *et al.*, 1989*b*; Budd *et al.*, 1992). However, low response rates of 10% or less were observed by others (Oldham *et al.*, 1992; Dillman *et al.*, 1993; Sparano *et al.*, 1993). The median response duration in these trials varied between 2 and 11 months, and the median survival was approximately 10 months (Lee *et al.*, 1989; Rosenberg *et al.*, 1989*b*; Oldham *et al.*, 1992; Dillman *et al.*, 1993; Sparano *et al.*, 1993). We achieved similar results.

We failed to confirm the ability of IFN-α to augment the effect of IL-2. This may have been due to suboptimal dose and schedule. Our patients received moderate doses of IL-2. In animal studies the efficacy of IL-2 is dose dependent without reaching a plateau below the maximum tolerated dose (Mule *et al.*, 1984). However, in trials using high-dose IL-2 (18 MIU $m^{-2}$ $day^{-1}$) given by continuous infusion in patients with metastatic melanoma inferior response rates were reported (Oldham *et al.*, 1992; Dillman *et al.*, 1993). An NCI Surgery Branch Study, administering high-dose bolus IL-2 (>30 MIU $m^{-2}$ $day^{-1}$) and IFN-α found the highest response rates (Rosenberg *et al.*, 1989*b*). On the other hand, the Extramural IL-2 Working Group, using identical dose, schedule and patient selection criteria, did not observe any evidence of enhanced response with the IL-2/IFN-α combination (Sparano *et al.*, 1993). In summary, a dose–response effect for IL-2 in the treatment of metastatic melanoma is not clear.

The side-effects we observed were of similar incidence and severity as reported previously (Lee *et al.*, 1989; Rosenberg *et al.*, 1989*b*; Budd *et al.*, 1992; Oldham *et al.*, 1992; Sparano *et al.*

al., 1993). Toxicity was manageable and patients tolerated the therapeutic regimen relatively well. However, cumulative fatigue made it impossible to give patients more than 13 cycles of therapy.

In conclusion, combined therapy with IL-2 and IFN-α in the described regimen has only moderate activity in the treatment of patients with metastatic melanoma. Further clinical trials have to be designed to improve therapeutic results.

**Acknowledgements**

The authors wish to thank Ms P Bos for preparing the manuscript.

### References

BRUNDA MJ, BELLANTONI D AND SULICH V. (1987). In vivo antitumour activity of combinations of interferon-α and interleukin-2 in a murine model. Correlation of efficacy with the induction of cytotoxic cells resembling natural killer cells. *Int. J. Cancer*, **40**, 365-371.

BUDD GT, OSGOOD B, BARNA B, BOYETT JM, FINKE J, MEDENDORP SV, MURTHY S, NOVAK C, SERGI J, TUBBS R AND BUKOWSKI RM. (1989). Phase I clinical trial of interleukin-2 and α-interferon: toxicity and immunologic effects. *Cancer Res.*, **49**, 6432-6436.

BUDD GT, MURTHY S, FINKE J, SERGI J, GIBSON V, MEDENDORP SV, BARNA B, BOYETT JM AND BUKOWSKI RM. (1992). Phase I trial of high-dose bolus interleukin-2 and interferon α-2a in patients with metastatic malignancy. *J. Clin. Oncol.*, **10**, 804-809.

CAMERON RB, MCINTOSH JK AND ROSENBERG SA. (1988). Synergistic antitumour effects of combination immunotherapy with recombinant interleukin-2 and a recombinant hybrid α-interferon in the treatment of established murine hepatic metastases. *Cancer Res.*, **48**, 5810-5817.

DILLMAN RO, CHURCH C, OLDHAM RK, WEST WH, SCHWARTZBERG L AND BIRCH R. (1993). Inpatient continuous-infusion interleukin-2 in 788 patients with cancer. The National Biotherapy Study Group experience. *Cancer*, **71**, 2358-2370.

IIGO M, SAKURAI J, TAMURA T, SAIJO N AND HOSHI A. (1988). In vivo anti-tumour activity of multiple injections of recombinant interleukin-2 alone and in combination with three different types of recombinant interferon on various syngeneic murine tumors. *Cancer Res.*, **48**, 260-264.

KIRKWOOD JM. (1991). Studies of interferons in the therapy of melanoma. *Semin. Oncol.*, **18** (Suppl. 7), 83-89.

LEE KH, TALPAZ M, ROTHBERG JM, MURRAY JL, PAPADOPOULOS N, PLAGER C, BENJAMIN R, LEVITT D AND GUTTERMAN J. (1989). Concomitant administration of recombinant human interleukin-2 and recombinant interferon α-2a in cancer patients: a phase I study. *J. Clin. Oncol.*, **7**, 1726-1732.

MULE JJ, SHU S, SCHWARZ SL AND ROSENBERG SA. (1984). Successful adoptive immunotherapy of established pulmonary metastases with lymphokine-activated killer cells and recombinant interleukin-2. *Science*, **225**, 1487-1489.

OLDHAM RK, BLUMENSCHEIN G, SCHWARTZBERG L, BIRCH R AND ARNOLD J. (1992). Combination biotherapy utilizing interleukin-2 and alpha interferon in patients with advanced cancer: a National Biotherapy Study Group trial. *Mol. Biother.*, **4**, 4-9.

PARKINSON DR, ABRAMS JS, WIERNIK PH, RAYNER AA, MARGOLIN KA, VAN ECHO DA, SZNOL M, DUTCHER JP, ARONSON FR, DOROSHOW JH, ATKINS MB AND HAWKINS MJ. (1990). Interleukin-2 therapy in patients with metastatic malignant melanoma: a phase II study. *J. Clin. Oncol.*, **8**, 1650-1656.

ROBINSON WA, MUGHAL TI, THOMAS MR JOHNSON M AND SPIEGEL RJ (1986). Treatment of metastatic malignant melanoma with recombinant interferon-alpha-2. *Immunobiology*, **172**, 275-282.

ROSENBERG SA, LOTZE MT, YANG JC, AEBERSOLD PM, LINEHAN WM, SEIPP CA AND WHITE DE (1989*a*). Experience with the use of high-dose interleukin-2 in the treatment of 652 cancer patients. *Ann. Surg.*, **210**, 474-485.

ROSENBERG SA, LOTZE MT, YANG JC, LINEHAN WM, SEIPP CA, CALABRO S, KARP SE, SHERRY RM, STEINBERG S AND WHITE DE. (1989*b*). Combination therapy with interleukin-2 and α-interferon for the treatment of patients with advanced cancer. *J.Clin. Oncol.*, **7**, 1863-1874.

ROSENBERG SA, LOTZE MT, YANG JC, TOPALIAN SL, CHANG AE, SCHWARTZENTRUBER DJ, AEBERSOLD P, LEITMAN S, MARSTON LINEHAN W, SEIPP CA, WHITE DE AND STEINBERG SM. (1993). Prospective randomized trial of high-dose interleukin-2 alone or in conjunction with lymphokine-activated killer cells for the treatment of patients with advanced cancer. *J. Natl Cancer Inst.*, **85**, 622-632.

SPARANO JA, FISHER RI, SUNDERLAND M, MARGOLIN KA, ERNEST ML, SZNOL M, ATKINS MB, DUTCHER JP, MICETICH KC, WEISS GR, DOROSHOW JH, ARONSON FR, RUBINSTEIN LV AND MEIR JW. (1993). Randomized phase III trial of treatment with high-dose interleukin-2 either alone or in combination with interferon alfa-2a in patients with advanced melanoma. *J. Clin. Oncol.*, **11**, 1969-1977.

WHITEHEAD RP, KOPECKY KJ, SAMSON MK, COSTANZI JJ, NATALE RB, FEUN LG, HERSH EM AND RINEHART JJ (1991). Phase II study of intravenous bolus recombinant interleukin-2 in advanced malignant melanoma. *J. Natl Cancer Inst.*, **83**, 1250-1252.

WHO (1979). *Handbook for Reporting Results of Cancer Treatment*. WHO: Geneva.


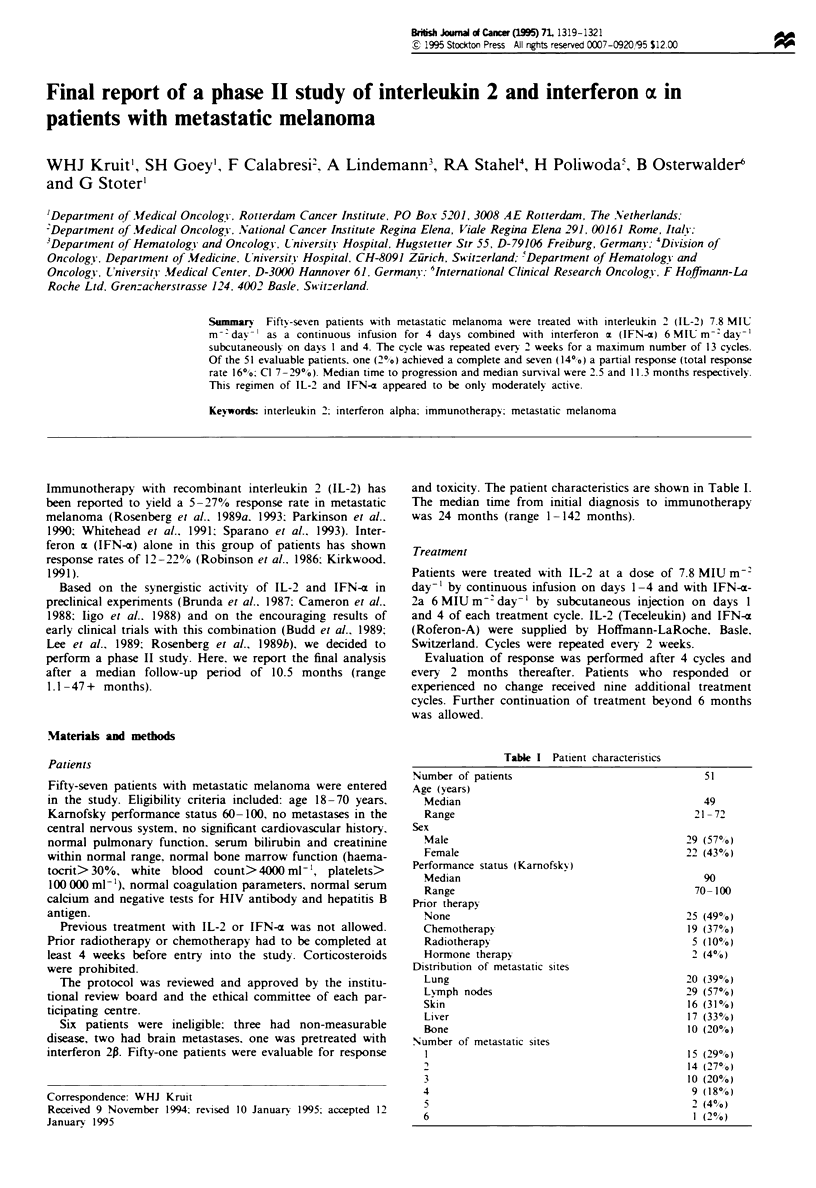

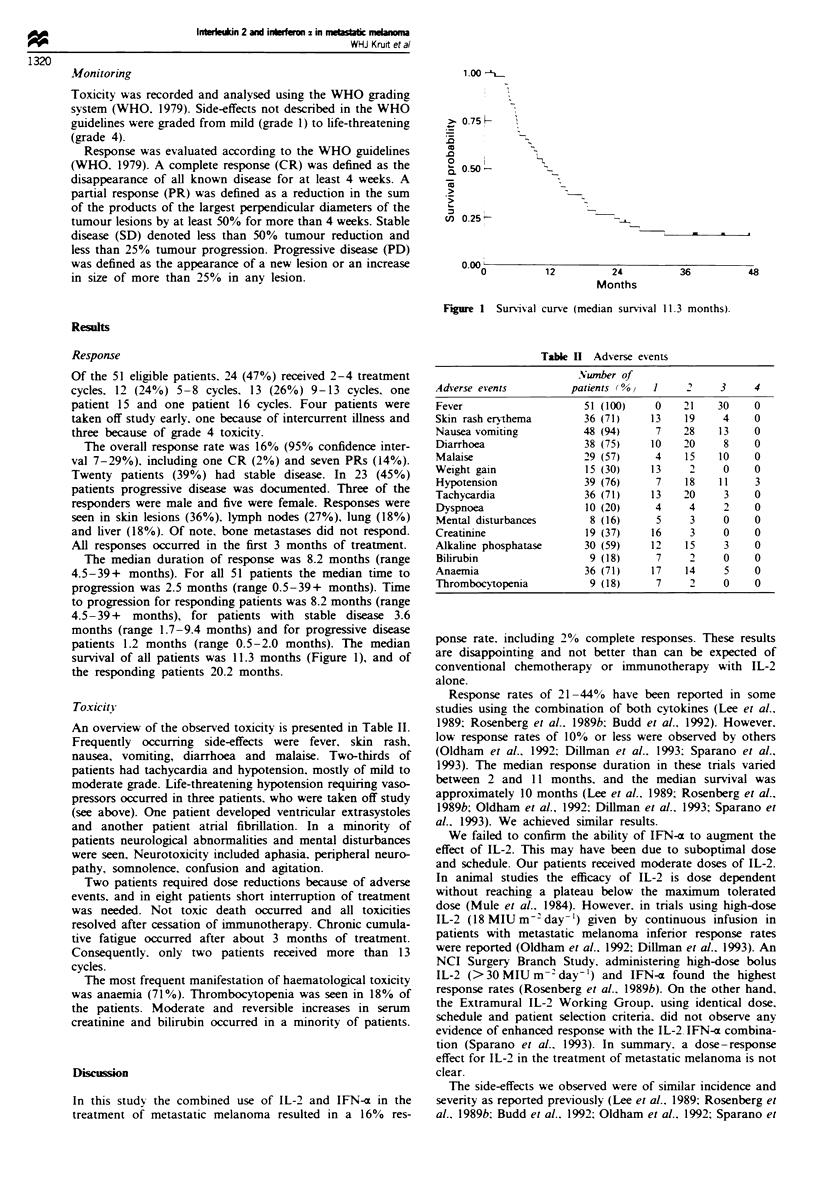

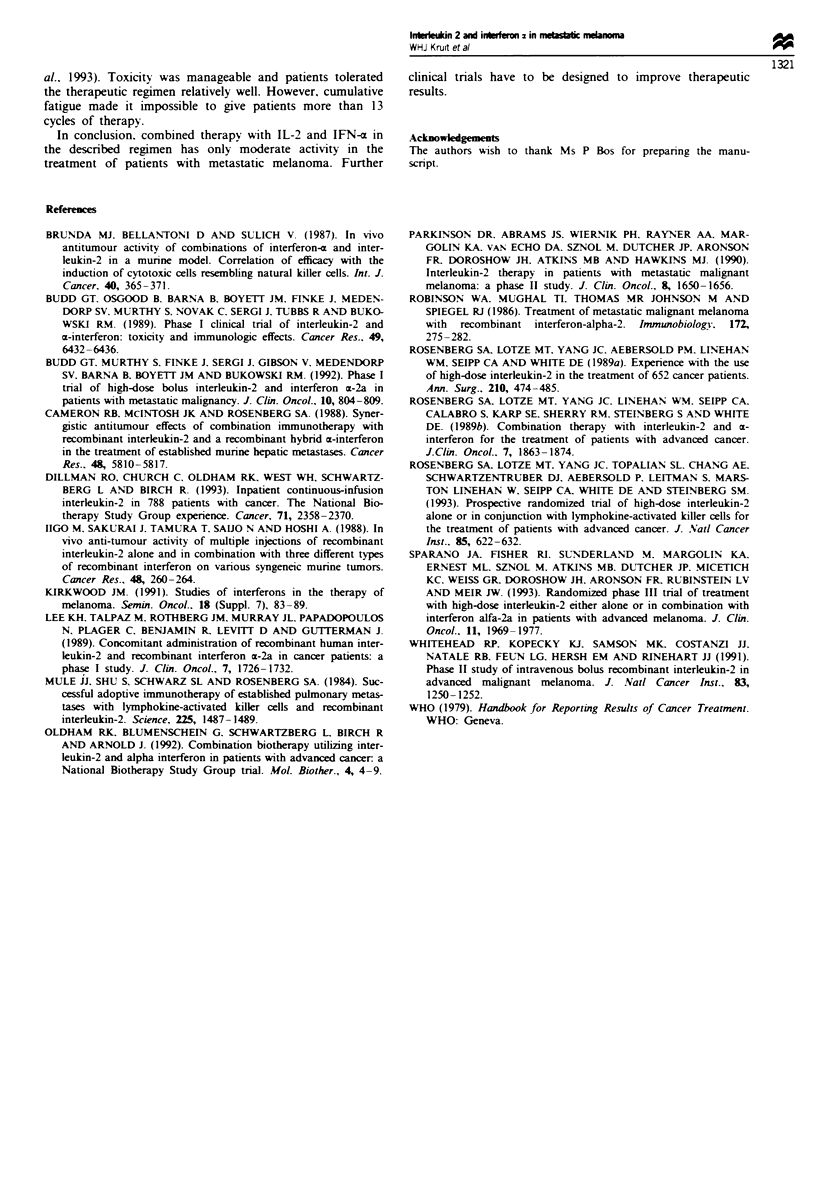


## References

[OCR_00361] Brunda M. J., Bellantoni D., Sulich V. (1987). In vivo anti-tumor activity of combinations of interferon alpha and interleukin-2 in a murine model. Correlation of efficacy with the induction of cytotoxic cells resembling natural killer cells.. Int J Cancer.

[OCR_00377] Budd G. T., Murthy S., Finke J., Sergi J., Gibson V., Medendorp S., Barna B., Boyett J., Bukowski R. M. (1992). Phase I trial of high-dose bolus interleukin-2 and interferon alfa-2a in patients with metastatic malignancy.. J Clin Oncol.

[OCR_00371] Budd G. T., Osgood B., Barna B., Boyett J. M., Finke J., Medendorp S. V., Murthy S., Novak C., Sergi J., Tubbs R. (1989). Phase I clinical trial of interleukin 2 and alpha-interferon: toxicity and immunologic effects.. Cancer Res.

[OCR_00382] Cameron R. B., McIntosh J. K., Rosenberg S. A. (1988). Synergistic antitumor effects of combination immunotherapy with recombinant interleukin-2 and a recombinant hybrid alpha-interferon in the treatment of established murine hepatic metastases.. Cancer Res.

[OCR_00390] Dillman R. O., Church C., Oldham R. K., West W. H., Schwartzberg L., Birch R. (1993). Inpatient continuous-infusion interleukin-2 in 788 patients with cancer. The National Biotherapy Study Group experience.. Cancer.

[OCR_00395] Iigo M., Sakurai M., Tamura T., Saijo N., Hoshi A. (1988). In vivo antitumor activity of multiple injections of recombinant interleukin 2, alone and in combination with three different types of recombinant interferon, on various syngeneic murine tumors.. Cancer Res.

[OCR_00407] Lee K. H., Talpaz M., Rothberg J. M., Murray J. L., Papadopoulos N., Plager C., Benjamin R., Levitt D., Gutterman J. (1989). Concomitant administration of recombinant human interleukin-2 and recombinant interferon alpha-2A in cancer patients: a phase I study.. J Clin Oncol.

[OCR_00413] Mulé J. J., Shu S., Schwarz S. L., Rosenberg S. A. (1984). Adoptive immunotherapy of established pulmonary metastases with LAK cells and recombinant interleukin-2.. Science.

[OCR_00423] Parkinson D. R., Abrams J. S., Wiernik P. H., Rayner A. A., Margolin K. A., Van Echo D. A., Sznol M., Dutcher J. P., Aronson F. R., Doroshow J. H. (1990). Interleukin-2 therapy in patients with metastatic malignant melanoma: a phase II study.. J Clin Oncol.

[OCR_00432] Robinson W. A., Mughal T. I., Thomas M. R., Johnson M., Spiegel R. J. (1986). Treatment of metastatic malignant melanoma with recombinant interferon alpha 2.. Immunobiology.

[OCR_00439] Rosenberg S. A., Lotze M. T., Yang J. C., Aebersold P. M., Linehan W. M., Seipp C. A., White D. E. (1989). Experience with the use of high-dose interleukin-2 in the treatment of 652 cancer patients.. Ann Surg.

[OCR_00442] Rosenberg S. A., Lotze M. T., Yang J. C., Linehan W. M., Seipp C., Calabro S., Karp S. E., Sherry R. M., Steinberg S., White D. E. (1989). Combination therapy with interleukin-2 and alpha-interferon for the treatment of patients with advanced cancer.. J Clin Oncol.

[OCR_00451] Rosenberg S. A., Lotze M. T., Yang J. C., Topalian S. L., Chang A. E., Schwartzentruber D. J., Aebersold P., Leitman S., Linehan W. M., Seipp C. A. (1993). Prospective randomized trial of high-dose interleukin-2 alone or in conjunction with lymphokine-activated killer cells for the treatment of patients with advanced cancer.. J Natl Cancer Inst.

[OCR_00458] Sparano J. A., Fisher R. I., Sunderland M., Margolin K., Ernest M. L., Sznol M., Atkins M. B., Dutcher J. P., Micetich K. C., Weiss G. R. (1993). Randomized phase III trial of treatment with high-dose interleukin-2 either alone or in combination with interferon alfa-2a in patients with advanced melanoma.. J Clin Oncol.

[OCR_00467] Whitehead R. P., Kopecky K. J., Samson M. K., Costanzi J. J., Natale R. B., Feun L. G., Hersh E. M., Rinehart J. J. (1991). Phase II study of intravenous bolus recombinant interleukin-2 in advanced malignant melanoma: Southwest Oncology Group study.. J Natl Cancer Inst.

